# The effect of family and friend support on physical activity through adolescence: a longitudinal study

**DOI:** 10.1186/s12966-015-0265-6

**Published:** 2015-08-20

**Authors:** Joanna L. Morrissey, Kathleen F. Janz, Elena M. Letuchy, Shelby L. Francis, Steven M. Levy

**Affiliations:** Kinesiology Department at California State, University-Monterey Bay, University Corp. 117C Seaside, Monterey, 93955 CA USA; Department of Health and Human Physiology and the Department of Epidemiology at the University of Iowa, 130 Field House, Iowa City, IA 52242 USA; Department of Epidemiology at the University of Iowa, 5205 Westlawn Building, Iowa City, IA 52242 USA; Department of Health and Human Physiology at the University of Iowa, E102 Field House, Iowa City, IA 52242 USA; Department of Preventive and Community Dentistry and Department of Epidemiology at the University of Iowa, N-328 Dental Science, Iowa City, IA 52242 USA

**Keywords:** Youth, Health, Accelerometer, Tracking, Physical activity

## Abstract

**Background:**

This study examined if family and friend support predicted adolescent physical activity (PA) across a five-year time span.

**Methods:**

The Iowa Bone Development Study collected objective measures of physical activity and self-report of physical activity psychosocial factors at ages 13 (*n* = 306), 15 (*n* = 356), and 17 yr (n = 317). Total moderate and vigorous-intensity PA (MVPA) and MVPA after 3 pm on weekdays (MVPA-PM Weekday) were measured using ActiGraph accelerometers. Family Support for PA and Friend Support for PA scales were measured using the Choices questionnaire. Models were adjusted for SES (mother’s education) and somatic maturity (Mirwald predictive equations for maturity offset). Spearman correlation coefficients examined tracking of scales at ages 13, 15 and 17. Logistic regression estimated the odds ratio for being in the lowest tertile of each scale at age 17 if in the lowest tertile at age 13. Linear mixed regression models investigated associations between these scales and MVPA outcomes over time.

**Results:**

Two- and five-year intra-variable tracking associations for Family Support and Friend Support scales were moderate (*r* = 0.32–0.58), except for the comparison between age 13 and age 17 Friend Support for girls, which resulted in a low association (*r* = 0.26). Boys and girls in the lowest tertile for support at age 13 were more likely to remain in the lowest tertile at age 17 compared to those in the middle and upper tertiles. The regression models indicated that when all other factors were held constant, an increase in family and/or friend support resulted in an increase in both MVPA outcomes

**Conclusions:**

From early to late adolescence, support for PA from the family and/or support from friends results in higher levels of total and discretionary MVPA. However, the importance of support in predicting MVPA decreased with age.

## Background

Habitual physical activity (PA) provides numerous health benefits [1, 2] yet, U.S. data from 2012 indicate that only 24.8 % of adolescents, aged 12–15 years, were participating in enough PA to meet the World Health Organization’s physical activity guidelines for children and adolescents of accumulating 60 min of at least moderate-intensity PA on a daily basis [3]. The recently published Physical Activity Guidelines for Americans Midcourse Report [2] presented emerging evidence and evidence-based practices that encourage and support PA in youth. The report focused on five settings in which youth PA interventions have been implemented and evaluated: schools, preschool/childcare centers, community, family/home, and primary care. Strategies that address adherence to and maintenance of PA are of particular importance during adolescence, when dramatic declines in PA rates occur [4, 5].

The Midcourse Report [2] notes the need for new health promotion strategies to encourage the development of lifelong motivation and involvement in PA among today’s youth. Within the family and home setting, the report suggests that family-based approaches have great potential to encourage and support youth PA, since PA-related habits, values, and beliefs are learned within the family environment [4]. In a systematic review of research examining the relationships between parental social support and youth PA, Beets and colleagues [6] reported that many studies identified positive associations among parental tangible (e.g., transportation, purchasing equipment, paying fees) and intangible (e.g., encouragement, praise, information) social support and youth PA. Exploring the direct and indirect relationship between parental social support and adolescent PA, Peterson and colleagues [7] found that parental instrumental social support (e.g., transportation) was directly related to females’ PA, while parental emotional social support (e.g., encouragement) was inversely related to females’ PA.

As adolescents become more autonomous from their parents they look more to their friends for behavioral and social cues [8]. Sirard and colleagues sought to explore the relationships between adolescents’ PA and screen time and their friends’ PA and screen time [9]. The researchers found that female PA and female screen time usage were associated with both their male and female friends’ PA, while male PA and male screen time usage were associated with their female friends’ PA. This work indicates the effect that friends have on adolescent lifestyle behaviors [8, 9].

Research suggests that adolescents accumulate greater activity levels outside the school environment [10, 11]. Cox and colleagues examined PA levels of school-aged children in both the school and out-of-school environments. Results showed that more steps were taken outside of the school environment (52.4 %) than during the school day (47.6 %) [10]. Additionally, when compared to the least active group, the most active children obtained a significantly higher proportion of their daily step count outside the school day. Similarly, Biddle and colleagues found that adolescents’ sport and exercise participation tend to peak in the early evening hours [12]. Thus, examining the specific time period of evening weekday MVPA is significant given adolescents are more active outside of the school setting.

The Midcourse Report [2] recommends that a ‘next step for research’ include conducting studies to examine which factors (e.g., specific components of family support) track throughout childhood and adolescence. By having a better understanding of how PA factors track through different lifespan periods, health promotion practitioners are better equipped to promote and deliver effective interventions during these unique periods of social and physiological development.

Using an objective measure of PA, subjective reporting of psychosocial factors associated with PA, and a five-year follow-up design, we examined how family support and friend support predicted adolescent PA levels at different ages (i.e., 13, 15, and 17 years) and levels of maturity. Specifically, we hypothesized that high levels of family support and friend support would predict high levels of total moderate and vigorous intensity PA (MVPA) and weekday evening MVPA. Additionally, we hypothesized that, as adolescents matured, family support would have a weaker association with MVPA and friend support would be more strongly associated with MVPA. Examining these relationships at various points during adolescence is crucial to tailoring effective PA strategies. As importantly, doing so with a longitudinal design reduces the confounding effects of variability in growth and maturation among adolescents.

## Methods

### Participants

Study participants were members of the Iowa Bone Development Study (IBDS), an ongoing, longitudinal study of bone health during childhood, adolescence, and young adulthood. They were a subset of Midwestern residents from a cohort of 890 families recruited from 1992–1995 to participate in the Iowa Fluoride Study. Additional information about the demographic characteristics of the original cohort participants and study design can be found elsewhere [13]. The IBDS used rolling admission and allowed Iowa Fluoride Study members to participate in any follow-up examinations or skip some examinations. IBDS cohort members participated in accelerometry assessments at ages 5, 8, 11, 13, 15, 17, and 19 years and completed a psychosocial questionnaire (described below) at ages 13, 15, and 17. Accelerometry assessment participation retention rate was high. For example, 88 % of participants with accelerometry assessments at age 13 (baseline for our current study) had assessments at age 15 or 17; 55 % of the age 13 participants had assessments at both age 15 and 17. The highest participation for the psychosocial questionnaire was at age 15 (75 % response rate). Overall, 519 participants had at least one accelerometry assessment with the psychosocial questionnaire completed at age 13 through 17.

This study required participants to have at least two assessments for longitudinal analysis, which reduced sample size to 401 participants (52 % girls). IBDS was approved by the University of Iowa Institutional Review Board for Human Subjects. Parents provided informed consent and minors provided assent.

### Choices questionnaire

IBDS uses the Choices Questionnaire as a measure of PA predisposing, reinforcing, and enabling factors [14]. These factors are measured via seven subscales (barrier self-efficacy, PA enjoyment, family support, friend support, perceived school climate, neighborhood safety and PA access). Participants rated all of their responses to the items on a 5-point scale ranging from 1 (disagree a lot) to 5 (agree a lot). Scales were calculated as mean value of responses for scale generating items. To be considered a valid scale value, no more than half of the items could be missing. All questions included in this questionnaire have been used in previous studies [15–21].

The current study focuses on the family and friend support scales. The family and friend support questions were initially developed and used as part of the Amherst Study; reliability and validity of these scales has been reported previously [22]. Cronbach’s alpha for these scales was 0.80 and 0.85, respectively. Prior pilot work using a subset of 52 adolescents (mean age of 13 years) in our cohort indicated internal consistency of 0.89 (Cronbach’s alpha) for the family support scale and 0.85 for the friend support scale.

Specific questions for the family and friend scales were also analyzed. The family support questions asked participants to think about ‘*the last 7 days’* and respond to: [1] *how often has a member of your household encouraged you to do physical activity or play sports?* (Family Encourage); [2] …*how often has a member of your household done a physical activity or played sports with you?* (Family Do); [3]…*how often has a member of your household provided transportation to a place where you can do physical activity or play sports?* (Family Transport); [4] *…how often has a member of your household watched you participate in a physical activity or sport?* (Family Watch); and [5] …*how often has a member of your household told you that you are doing well in a physical activity or sport?* (Family Told).

The friend support questions asked participants to think about ‘*the last 7 days’* and respond to: [1] … *how often did you encourage your friends to do physical activity or play sports?* (You Encourage); [2] … *how often did your friends encourage you to do physical activity or play sports?* (Friend Encourage); [3]… *how often did your friends do a physical activity or play sports with you?* (Friend Do); and [4] *… how often did your friends tell you that you were doing well in a physical activity or sport?* (Friend Told). Item 1 (You Encourage) was not included in the analysis since it asks participants how they provide support to their friends rather than how they perceive their friends to provide support to them.

### Physical activity

Physical activity was measured using ActiGraph accelerometer model number 7164 at age 13. Due to the discontinuation of models and the development of new models, model GT1M was used at age 15, and models GT3X and GT3X+ were used at age 17. Previous research has shown comparability between these monitors [23] and that they are valid and reliable for monitoring PA in field settings [24–28]. In the accelerometer data reduction process, a period of 60 or more consecutive minutes of zero accelerometer counts (with an allowance for two non zero interruptions) was considered as not wearing the monitor and invalid data. The low frequency extension (LFE) option was not used at any data collection point.

Older children have previously been shown to have less stable intra-class correlation coefficients in activity monitored PA compared to younger children, indicating the need for an additional day of monitoring [29]. Therefore, participants were asked to wear the monitor for five consecutive days, including both weekend days. To be included in the analyses, participants must have obtained at least three valid days of wear for each measurement period, around 5.6 % of records were excluded due to lower than 3 days of wear combining age 13, 15, and 17 assessments. A valid day consisted of wearing the monitor for at least 10 h per day. Using the Spearman–Brown prophecy formula, this corresponds to a 60 % reliability coefficient [30]. To reduce seasonal effects, PA was only measured during the autumn months. ActiGraph movement counts were collected in one-minute epochs at age 13, five-second epochs for age 15, and continuous, raw data for age 17. The five-second epochs and raw data were later re-integrated to one minute epochs to maintain consistency with the data collected at age 13.

The PA variables of interest were total time in MVPA (minutes) and time in MVPA after 3 pm on weekdays (MVPA-PM Weekday; minutes). Participants were required to have two valid days of wear for the MVPA-PM Weekday variable and three valid days of wear for the total MVPA variable. Mean values for these variables were obtained from all minutes of all valid days of wear. Based on a comparison of five independently developed sets of cut-points, Trost et al. [31] recommend that researchers use the cut-points developed by Evenson and colleagues [32]. The Evenson cut-point for MVPA is ≥ 2,296 counts per minute. This cut-point has been validated for youth ages 5–15 years using area-under-the-Receiver-Operating-Characteristic-Curve (ROC-AUC) where an area of 1 indicates perfect classification and an area of 0.5 represents an absence of classification accuracy [33]. The MVPA cut-point has been shown to exhibit fair (ROC-AUC = 0.74) classification accuracy.

### Additional variables for adjusting models

Co-variables for model adjustments included social economic status (SES), height, weight, and somatic maturity. Maternal education (Mother’s Ed) was collected as a part of the parents’ health history questionnaire at a clinical examination and dichotomized as high school (some or complete) or college (some college, 2-year degree, bachelor degree, graduate/professional degree). Mother’s Ed served as an indicator of SES since maternal education is a strong determinant of parental employment and income [34]. Also, at a clinical visit, research nurses trained in anthropometry measured participants’ height and sitting height using a Harpenden stadiometer (Holtain, Crymych, UK) and weight using a Healthometer physician’s scale (Continental, Bridgeview, IL). Standing and sitting height were used in prediction equations established by Mirwald and colleagues [35] to calculate Maturity Offset (years from peak height velocity (PHV)), which served as an estimate of participants’ somatic maturity. The method of Mirwald has been validated in white Canadian children and adolescents (R^2^ = 0.91–0.92, SEE = 0.49–0.50) [35].

### Statistical analysis

Sex-specific descriptive statistics (mean and SD) were calculated for the Family, and Friend scales, MVPA and MVPA-PM Weekday characteristics of the participants, and Mother’s Ed. Student’s t-tests and chi-square tests were used to examine sex differences. One-sample t-tests were used to test for differences in the Family Support and Friend Support scales from age 13 to age 17. Simple linear regression models with Maturity Offset as the independent variable and each of the two scales as the outcome were used to test for trend of each scale over time. Spearman correlation coefficients were used to examine tracking of Family Support and Friend Support scales from age 13 to age 17. Logistic regression determined the odds ratio of being in the lowest tertile of each scale at age 17 if in the lowest tertile at age 13, compared to the middle and upper tertiles.

Linear mixed regression models were used to investigate the associations between the two scales and MVPA and MVPA-PM Weekday over time. These models were adjusted for Sex and Mother’s Ed. Interaction terms for the scales and Maturity Offset were included in the models to investigate whether the associations changed over time. Two-way interactions with Sex were also included. Linear and squared terms for Maturity Offset were included to describe the average trajectory of PA over time and during maturation. Independent variables (*p* < 0.1) were kept in the models with the assumption that they may affect results. Additional models using the individual Family and Friend questions were also run. Mixed models with heterogeneous autoregressive variance-covariance residual structure grouping by Sex was selected as the best-fitting model based on the Akaike’s Information Criterion (AIC). Statistical significance was set at *p* < 0.05 for all analyses, which were conducted using SAS version 9.2.

A total of 118 participants were excluded due to only one assessment with both accelerometry and Choices data available (age 13 = 55 cases, age 15 = 25 cases, age 17 = 38 cases). There were no statistically significant differences between IDBS participants excluded and included in the analysis in age, somatic maturity, body size measures, MVPA, or Family Support scale (t-tests p-value > 0.15) at any assessment, but excluded participants had slightly higher Friend Support scale scores at age 13 (3.4 vs. 3.1, *t*-test p-value = 0.046) and 17 (3.0 vs. 2.6, *t*-test *p*-value = 0.034).

Table 1 describes participants. As expected, a higher percentage of girls had reached significantly more somatic maturity than the boys at each measurement period (*p* < 0.01). The boys were significantly taller and heavier than the girls at ages 15 and 17 (*p* < 0.01). The boys participated in significantly more minutes of MVPA and MVPA-PM Weekday than the girls at all measurement periods (*p* < 0.01). At least 88.8 % of each subgroup of the participants’ mothers had at least some college education. The trend analysis using a simple linear regression with maturity offset as the independent variable indicated that, with increasing maturity, the Family Support and Friend Support scale responses became more negative, i.e., scores decreased, meaning this variable was less important as a determinant of PA (Family Support: β = −0.16, SE = 0.01, *p* < 0.01; Friend Support: β = −0.09, SE = 0.01, *p* < 0.01). This trend can be seen in the mean scale scores in Table 1.

**Table 1:** Characteristics of participants

	Age 13 yr		Age 15 yr		Age 17 yr	
	Boys (*n* = 149)	Girls (*n* = 157)	Boys (*n* = 177)	Girls (*n* = 179)	Boys (*n* = 158)	Girls (*n* = 159)
Chronological Age (yr)	13.0 (0.2)	13.1 (0.3)	15.0 (0.3)	15.1 (0.3)	17.2 (0.5)	17.2 (0.5)
Maturity Offset^a^ (yr)	−0.7 (0.8)**	1.3 (0.7)	1.4 (0.8)**	3.3 (0.7)	3.5 (0.9)**	5.4 (0.8)
Height (cm)^b^	162.6 (9.3)	160.9 (6.8)	175.4 (8.1)**	164.5 (6.3)	178.7 (8.0)**	165.7 (6.8)
Weight (kg)^b^	57.9 (15.4)	56.3 (14.4)	70.8 (16.2)**	61.8 (14.4)	79.5 (19.3)**	66.9 (16.4)
Family Support (1–5)	3.1 (0.9)*	2.9 (0.9)	2.8 (0.9)*	2.6 (0.9)	2.2 (0.9)	2.1 (0.9)
Family Encourage	3.5 (1.2)*	3.2 (1.2)	3.4 (1.2)**	3.1 (1.3)	2.7 (1.3)	2.5 (1.3)
Family Do	2.8 (1.2)**	2.4 (1.2)	2.4 (1.2)**	2.0 (1.1)	1.9 (1.2)	1.8 (1.1)
Family Transport	3.2 (1.4)	3.3 (1.4)	3.1 (1.4)	3.0 (1.4)	1.9 (1.5)	1.8 (1.3)
Family Watch	2.8 (1.2)	2.6 (1.2)	2.3 (1.1)	2.3 (1.2)	1.9 (1.1)	1.9 (1.0)
Family Told	3.4 (1.3)**	3.0 (1.1)	3.0 (1.3)	2.8 (1.3)	2.4 (1.4)	2.3 (1.3)
Friend Support (1–5)	3.2 (0.9)	3.0 (1.0)	2.9 (1.1)	2.8 (1.1)	2.8 (1.1)	2.5 (1.1)
Friend Encourage	2.9 (1.3)	2.7 (1.2)	2.8 (1.3)**	2.5 (1.2)	2.7 (1.2)*	2.4 (1.2)
Friend Do	3.7 (1.1)	3.6 (1.2)	3.4 (1.3)	3.3 (1.3)	3.2 (1.3)	2.9 (1.4)
Friend Told	2.8 (1.2)	2.8 (1.3)	2.6 (1.3)	2.7 (1.3)	2.4 (1.3)	2.3 (1.3)
Time accelerometer worn (min/day)	779.5 (54.1)	787.1 (60.2)	804.6 (59.3)	799.5 (63.0)	800.4 (62.2)	812.5 (69.9)
MVPA^c^ (min/day)	52.0 (21.7)**	35.1 (20.5)	39.1 (20.3)**	26.2 (17.2)	37.7 (21.7)**	23.8 (15.0)
MVPA- PM Weekday^cd^ (min/day)	30.3 (19.4)**	22.0 (15.8)	24.1 (18.9)**	15.6 (14.9)	20.6 (17.1)**	15.0 (14.1)
Transformed MVPA^c^	7.4 (1.4)**	6.1 (1.7)	6.5 (1.6)**	5.3 (1.7)	6.3 (1.7)**	5.1 (1.5)
Transformed MVPA- PM Weekday^cd^	5.1 (1.5)**	4.3 (1.6)	4.5 (1.6)**	3.5 (1.8)	4.1 (1.8)**	3.5 (1.7)
	N (%)	N (%)	N (%)	N (%)	N (%)	N (%)
Mother’s Ed (hs vs college)^e^	136 (91.3)	143 (91.1)	161 (91.0)	159 (88.8)	143 (90.5)	145 (91.2)

*Note.* Values are presented as mean (SD)

*Note. MVPA* moderate and vigorous-intensity physical activity

^a^Maturity Offset calculated in years from age at peak height velocity

^b^Reported for all non-missing data, only participants with clinical exam DXA scans were measured (13-20 % missing depending on assessment and sex)

^c^Untransformed and Box-Cox transformed minutes of MVPA reported; transformed values used in all inferential analyses

^d^MVPA- PM Weekday = number of minutes of MVPA occurring after 3 pm on weekdays only

^e^Mother’s Ed dichotomized as High School (some or complete) vs. College (some college, 2-year degree, bachelor degree, graduate/professional degree)

*p < 0.05, **p < 0.01 Student’s *t*-test (or chi-square test for Mother’s Ed and Dichotomized Enjoyment) of boys vs. girls

**Fig. 1:**
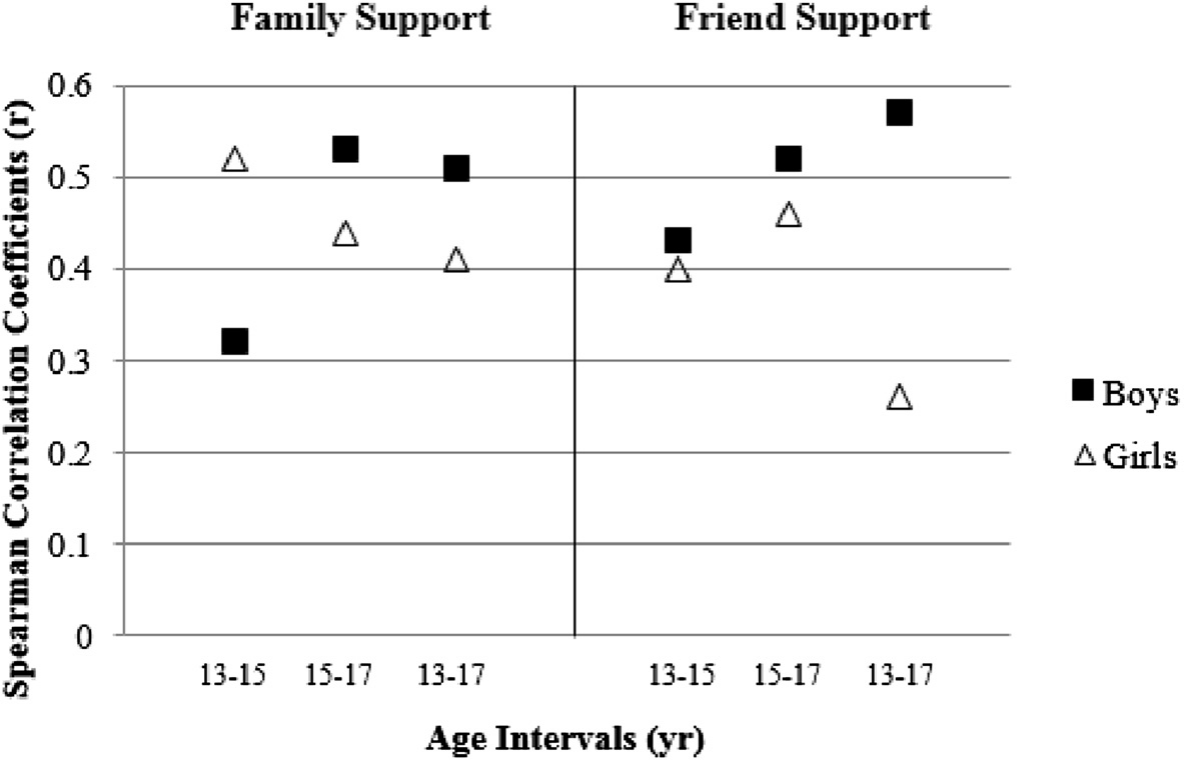
Spearman correlation coefficients by sex for each questionnaire scale. *Note.* All coefficients statistically significant (*p* < 0.05)

The Spearman correlation coefficients used to examine tracking of the scales are shown in Fig. 1. As suggested by Malina [36], correlations were interpreted as follows: < 0.30 low, 0.30 to 0.60 moderate, and > 0.60 moderately high. All comparisons resulted in moderate associations, except for the comparison of Friend Support for girls between the age 13 and age 17, which resulted in a low association (*r* = 0.26). This low association indicates there is less of a relationship between girls’ perceptions of Friend Support on PA when comparing Friend Support at age 13 and 17 than at any other age comparison.

Logistic regression was used to estimate the odds of remaining in the lowest tertile of each scale at age 17 if already in the lowest tertile at age 13, compared to the middle and upper tertile at age 13 (Table 2). For both scales, boys and girls in the lowest tertile at age 13 were significantly and substantially more likely to remain in the lowest tertile at age 17 compared to those in the middle and upper tertile.

**Table 2:** Logistic regression: Odds ratio for staying in the lowest scale tertile at age 17 if in lowest tertile at age 13, compared to middle and upper tertile at age 13

	Boys	Girls
	OR (95 % CI)	OR (95 % CI)
Family Support	6.9 (2.6, 18.3)	3.7 (1.4, 9.4)
Friend Support	13.8 (4.8, 40.2)	3.7 (1.4, 9.7)

*Note.* Variables dichotomized as 1 = lowest tertile of scale and 0 = middle/upper tertiles combined

*Note. OR* odds ratio, *CI* confidence interval

*Note.* Mean for lowest tertile for boys’ Family Support at age 17 was 1.19 and mean for Friend Support was 1.50. Mean for lowest tertile for girls’ Family Support at age 17 was 1.15 and mean for Friend Support was 1.28

Regression models for MVPA are presented in Table 3. Sex was a significant predictor of MVPA (β =1.574, *p* < 0.001); boys had more MVPA than girls when all other factors were held constant. Overall, an increase in Family Support resulted in an increase in MVPA if all other factors were held constant (β = 0.667, *p* < 0.001). However, the significant Maturity Offset * Family Support interaction term (β = −0.080, *p* < 0.01) indicated that, as participants matured, the influence of Family Support was reduced, resulting in smaller increases in MVPA. The Family Support * Sex interaction term (β = −0.298, *p* <0.05) indicated that Family Support was more influential on MVPA for girls than for boys. An increase in Friend Support also resulted in an increase in MVPA if all other factors were held constant, however, this effect was smaller than for Family Support (Friend Support β = 0.267 versus Family Support β = 0.677).

**Table 3:** Mixed multivariate linear regression models of MVPA and MVPA- PM Weekday for boys and girls combined as predicted by family support and friend support

	Effect	β	SE	P-Value
MVPA	Intercept	3.233	0.397	<0.001
	Maturity Offset	−0.077	0.098	0.434
	Maturity Offset* Maturity Offset	0.026	0.009	0.005
	Sex (ref = girl)	1.574	0.335	<0.001
	Family Support	0.677	0.128	<0.001
	Maturity Offset* Family Support	−0.080	0.028	0.004
	Family Support* Sex (ref = girl)	−0.298	0.118	0.012
	Friend Support	0.267	0.055	<0.001
	Mother’s Ed	0.355	0.192	0.065
MVPA-	Intercept	2.070	0.274	<0.001
PM Weekday	Maturity Offset	−0.251	0.050	<0.001
	Maturity Offset* Maturity Offset	0.026	0.009	0.004
	Sex (ref = girl)	0.385	0.126	0.002
	Family Support	0.315	0.065	<0.001
	Friend Support	0.352	0.056	<0.001
	Mother’s Ed	0.455	0.194	0.019

*Note. β* regression parameter estimate, *MVPA* moderate through vigorous-intensity PA

*Note.* Box-Cox transformed MVPA minutes used for inferential analyses. *N* = 401 (207 girls, 194 boys) participants with ≥2 assessments were used for modeling (979 records)

Regression models for MVPA-PM Weekday are also presented in Table 3 and were similar to the results for overall MVPA. Like the MVPA results, Sex, Family Support, and Friend Support were all significant predictors of MVPA-PM Weekday. Contrary to the MVPA results, Maturity Offset was a significant predictor of MVPA-PM Weekday, which indicated that, as participants matured, they participated in less MVPA-PM Weekday. Also, Friend Support resulted in a slightly larger increase in MVPA-PM Weekday (Friend Support β = 0.352 versus Family Support β = 0.315). Finally, Mother’s Ed was a significant predictor of MVPA-PM Weekday (β = 0.455, *p* < 0.05).

Additional regression models for MVPA investigating the individual questions from the Family Support and Friend Support scales are shown in Table 4. An increase in Maturity Offset was the only variable that resulted in a significant decrease in MVPA. Sex (being a boy), Family Encourage, Family Watch, and Friend Do all resulted in increases in MVPA. Family Do, Family Transport, Family Told, Friend Encourage, and Friend Told never reached the level necessary (*p* < 0.1) to enter the models.

**Table 4:** Mixed multivariate linear regression models of MVPA and MVPA- PM Weekday for boys and girls combined as predicted by family support and friend support subscales

	Effect	β	SE	P-Value
MVPA	Intercept	3.943	0.271	<0.001
	Maturity Offset	−0.292	0.048	<0.001
	Maturity Offset* Maturity Offset	0.029	0.009	<0.001
	Sex (ref = girl)	0.819	0.126	<0.001
	Family Encourage	0.119	0.041	0.004
	Family Watch	0.177	0.046	0.001
	Friend Do	0.309	0.041	<0.001
	Mother’s Ed	0.381	0.193	0.049
MVPA-	Intercept	1.945	0.263	<0.001
PM Weekday	Maturity Offset	−0.219	0.050	<0.001
	Maturity Offset* Maturity Offset	0.021	0.009	0.019
	Sex (ref = girl)	0.435	0.123	<0.001
	Family Do	0.078	0.045	0.085
	Family Watch	0.207	0.048	<0.001
	Friend Do	0.377	0.042	<0.001
	Mother’s Ed	0.450	0.192	0.020

*Note.* β, regression parameter estimate; MVPA, moderate through vigorous-intensity PA

*Note.* Box-Cox transformed MVPA minutes used for inferential analyses. *N* = 401 (207 girls, 194 boys) participants with ≥ 2 assessments were used for modeling (979 records)

Additional regression models for MVPA-PM Weekday investigating the specific questions from the Family Support and Friend Support scales are also shown in Table 4, with results very similar to MVPA. Again, an increase in Maturity Offset was the only variable that resulted in a significant decrease in MVPA-PM Weekday. Sex (being a boy), Family Watch, and Friend Do all resulted in increases in MVPA-PM Weekday. The only difference for MVPA-PM Weekday from MVPA was that Family Encourage, in addition to Family Do, Family Transport, Family Told, Friend Encourage, and Friend Told, did not reach the level necessary (*p* < 0.1) to enter the model.

## Discussion

This study is among the first to track the adolescent PA psychosocial determinant of support and associate it with objectively-measured PA. We show that, holding all other factors constant, the presence of family support and/or the presence of friend support results in higher levels of total MVPA and weekday evening MVPA from ages 13–17 years.

### Sex differences

Our results show that boys indicated higher parent support and friend support than girls. Thus, boys perceive family and friends to take a more active role in supporting in their PA efforts than do girls. Previous studies that have examined sex differences in psychosocial determinants (motivation, enjoyment, self-efficacy) related to PA have shown inconsistent findings. In general, previous research suggests a stronger relationship between PA and psychosocial factors, like enjoyment, support and motivation for boys than girls [37, 38]. For example, Wu and colleagues examined self-efficacy as a mediator between social influences (parent and peer emotional support, modeling, norms) and adolescent PA, and found the pathway between peer social influences and adolescent self-efficacy to be stronger for boys than girls [37]. However, Ferrer-Caja and Weiss found the relationship between intrinsic motivation and motivated PA behaviors were similar for boys and girls in physical education classes and thus, did not find support for sex-specific models [39]. The Midcourse Report [2] encourages the development of strategies to cultivate lifelong PA motivation and PA involvement among today’s youth. Health promotion practitioners should take extra care to design inclusive strategies, messages, and programs targeting both boys and girls. Added effort and attention to increasing the presence of support for PA in adolescent girl physical activities and sport programs could help young girls to cultivate a lifelong involvement in PA, which would meet an objective of the Midcourse Report.

### Tracking support determinants

While studies investigating tracking of support determinants with an objective measure of PA in adolescents (over a 5-year period) could not be found in the existing literature, De Bourdeaudhuij and colleagues [40] investigated the tracking of self-reported PA and psychosocial variables including support over a 7-year period in young adults. De Bourdeaudhuij et al. found correlations of 0.25 and 0.24 for social support from family and friends, respectively. Aside from the friend support comparison between age 13 and age 17 for girls (which was low), our findings resulted in moderate associations. Our odds ratio findings are relatively high and show substantial predictability of low levels of family support and friend support remaining low through the teenage years. The stable tracking of low support throughout adolescence suggests youth who need more support can be identified early and interventions could potentially be tailored toward such groups.

### The effect of family and friends on pa

We found that the presence of family support and/or friend support resulted in higher levels of total MVPA and weekday evening MVPA for our participants. In particular, participants who had friends that participated in PA with them had higher levels of total MVPA and weekday evening MVPA. Humbert and colleagues examined factors influencing youth PA in grades 7–12 and found that friends and adults played a role in adolescents’ PA participation [41]. In relation to friend support, adolescents participated in PA to be active with friends, meet new people, make new friends, and have fun with their current friends. Participants also stressed the importance of support from adults: to supervise and facilitate PA, be an active participant, and to have fun with adults [41]. In a study of youth soccer players (aged 10–14 years), Ulrich and Smith found that peer and parent relationships along with close social relationships had the greatest impact on continued sport participation [42]. Participants with higher perceived peer acceptance, friendship quality, and soccer competence were more likely to continue on with the sport [42]. Family, in particular parents, has been consistently and strongly linked with youth’s PA and sport involvement [43–47]. Our findings support the existing literature that cites the importance of family and friend support on adolescent PA. We add to the literature by assessing the trending influences of family support and friend support on adolescent PA levels using an objective measure of PA. Based on our findings, proactive strategies that focus on the inclusion of family and friends in early adolescence may sustain the presence of social support throughout the adolescent years and thus, cultivate a habit of participating in PA throughout the lifespan.

A limitation of our work is that it is unknown if family and friends are providing less support as adolescents mature, or if adolescents are placing less value on support as they mature. The use of multiple accelerometer models (due to discontinuation of old models and development of new models) was also a limitation since different models may yield variable output data. While newer accelerometer models included additional functions, like the low frequency extension (LFE) option which is designed to detect lower amplitude movements and possibly reduce measurement error [48, 49], we did not use the LFE option in an effort to remain consistent with previous IBDS method design and data collection periods. An additional limitation was an inadequate representation of minorities and adolescents from low SES households, due to the use of a Midwest, mostly rural, convenience sample. While our sample was largely non-Hispanic white (95 % of participants), this percentage is similar to the population demographics of Iowa (87.5 % of Iowans self-identify as non-Hispanic white). White Americans contribute the highest proportion of the population in the Midwest at 85 % according to the Population Estimates Program [50]. Non-Hispanic Whites make up 79 % of the Midwest’s population, which is the highest ratio of any region [50], but is similar to our sample demographics.

## Conclusions

This study advances the current literature by using three measurement periods (i.e., 13, 15, and 17 years of age) to track and assess the trending influences of family support and friend support on adolescent PA levels using an objective measure of PA. Importantly, we established that family and friend support continue to be important predictors of PA during the adolescent years. However, younger adolescents (i.e., 13 years of age) who receive little support from family and friends continue on this trajectory of low PA support into later adolescence (i.e., 17 years of age). Accordingly, family-based interventions should target younger adolescents to cultivate a supportive environment early on so such support is established and maintained through the adolescent time period. Additionally, interventions should include peer relationship-building activities to foster a supportive peer environment within the PA setting.

## Abbreviations

AIC: Akaike’s Information Criterion
β: Regression parameter estimate
CI: Confidence interval
Mother’s Ed: Maternal education
MVPA: Moderate and vigorous intensity physical activity
MVPA- PM Weekday: Number of minutes of MVPA occurring after 3 pm on weekdays only
OR: Odds ratio
PA: Physical activity
SD: Standard deviation
SES: Social economic status
PHV: Years from peak height velocity
ROC-AUC: Area-under-the-Receiver-Operating-Characteristic-Curve
